# A National School Health Campaign in Lebanon on Children Aged between 3 and 12 Years Old: Concordance Level between Parents’ Reports and Medical Visit Findings about Physical and Mental Health

**DOI:** 10.3390/children11020214

**Published:** 2024-02-07

**Authors:** Léa Tahan, Peter Habchy, Charbel Moussi, Tia Khadra, Melissa Jawich, Alain Njeim, Ogarite Kattan, Leila Abou Habib, Wassim El Bitar, Béchara El Asmar, Mirna N. Chahine

**Affiliations:** 1 Faculty of Medical Sciences, Lebanese University, Hadath P.O. Box 3, Lebanon; lea.tahan@st.ul.edu.lb (L.T.); p.habchy@st.ul.edu.lb (P.H.); c.moussa@st.ul.edu.lb (C.M.); tia.khadra@st.ul.edu.lb (T.K.); melissa.jawich@st.ul.edu.lb (M.J.); a.njeim@st.ul.edu.lb (A.N.); o.kattan@st.ul.edu.lb (O.K.); 2 Lebanese Association of the Knights of Malta (Order of Malta Lebanon), Vanlian Bldg, 6th Fl. City Rama Str. Dekwaneh, Beirut P.O. Box 11-4286, Lebanon; leila.sebeel@yahoo.com (L.A.H.); wassim.elbitar@chirec.be (W.E.B.); bechara.elasmar@hdf.usj.edu.lb (B.E.A.); 3 Department of Pediatrics, Bellevue Medical Center University Hospital, Mansourieh P.O. Box 295, Lebanon; 4 Faculty of Medicine, Saint Joseph University, Beirut P.O. Box 17-5208, Lebanon; 5 Department of Cardiology, Hotel-Dieu de France Hospital, Achrafieh, Beirut P.O. Box 11-5190, Lebanon; 6 Basic Sciences Department, Faculty of Medical Sciences, Lebanese University, Hadath P.O. Box 3, Lebanon; 7 Foundation-Medical Research Institutes (F-MRI®), Achrafieh, Beirut P.O. Box 64, Lebanon; 8 Foundation-Medical Research Institutes (F-MRI®), 1211 Geneva, Switzerland

**Keywords:** children, parents, Lebanon, schools, interventions, screening, mental health, concordance, mismatch

## Abstract

A school’s commitment to promoting health extends beyond students’ efforts to encompass parental involvement and must recognize the critical role of parents in enhancing overall student well-being. This study, conducted in 27 schools across Lebanon, assessed parents’ awareness of their children’s physical and mental health. A school health campaign involved direct medical interventions on 7184 students, followed by phone interviews with 3880 parents to compare their responses with the medical findings. Discordances ranged from extreme mismatches (≥50%) to mild mismatches (<15%), with notable disparities in incomplete vaccination (67.8%), BMI (59%), and mental health indicators (expressions of sadness (69.1%), loneliness (61.0%), and anxiety (53.4%)). Factors such as school type, child’s age, governorate, family income, parents’ occupation, education level, and marital status significantly influenced discordance rates. Notably, mental health aspects exhibited higher disparities, emphasizing the need for improved communication between parents, physicians, and children. Bridging these gaps could empower parents with better knowledge, fostering environments conducive to lifelong healthy behaviors in children. The study underscores the urgency for enhanced communication strategies to bridge discrepancies and ensure a more comprehensive understanding of children’s physical and mental well-being.

## 1. Introduction

Leading health indicators, including adopting healthy behaviors and being in a healthy social environment, are strongly correlated with health literacy (HL). Health literacy, defined as the ability to obtain, process, and understand health information to make informed decisions, plays a pivotal role in achieving and maintaining positive health outcomes. Given that they are accountable for both their children’s health and their own, parents are a crucial subgroup within the broader adult population. As caregivers, they must be equipped with the knowledge and skills necessary to prevent and address health problems in their children, since the latter are dependent on their parents in this regard. Without changes aimed at improving parents’ ability to fulfill this role, children may experience negative health outcomes due to inadequate care [1,2].

The significance of parental health literacy as a moderator of child health disparities was highlighted in a study by Sanders et al. It is important to mention that parental health literacy extends to the parents’ capacity to understand and apply health-related information for the well-being of their offspring. The research established a correlation between low parental HL and a limited set of health factors. Several adverse consequences emerged, including increased risk of exposure to secondhand tobacco smoke, unmet healthcare needs for children, poor childhood nutrition, and incorrect perceptions of their weight [3]. This idea was further validated by a cross-sectional survey that was carried out in 28 elementary and secondary schools in Germany, where 45.8% of the participating parents displayed unsatisfactory HL [4].

It is important to note that general HL is not synonymous with the health literacy of parents’ own children. As shown in a previous Lebanese study [5], parents’ knowledge about pediatric general health was assessed; however, the assessment of personalized knowledge of parents regarding their own children’s health was missing. Actually, in this study, students’ parents participated in a workshop related to parent–child communication skills as well as physical activities and eating habits. The results showed a significant increase in the general knowledge of parents after attending the awareness sessions regarding communications skills and healthy lifestyles-related knowledge.

Improving HL among parents is undoubtedly essential, but it is equally important to acknowledge the critical role that schools play in promoting medical knowledge. Therefore, implementing school-based health campaigns is crucial for improving the health and well-being of children. A review of the history of health programs in schools shows that the latter has been called to play a significant role in addressing social and health issues [6]. A crucial point to note is that a school that promotes health incorporates not just the students’ efforts but also those of parents to enhance the students’ well-being [7]. The results from a national project support school health in disadvantaged rural areas in Bekaa and Southern Lebanon [5]. Intervention campaigns implemented in schools, which actively engage parents, have been observed to yield greater success rates [8]. The preceding idea can help children acquire healthy habits in a good and thoughtful way. Furthermore, it can foster a stronger connection between them. This bond is essential as it enables parents to be more cognizant and knowledgeable about their children’s health and behaviors.

As a matter of fact, studies have looked at how much agreement there is between children and their parents on specific issues like quality of life, pain, and psychosocial functioning. In “Agreements and disagreements between children and their parents in health-related assessments” [9], the authors systematically reviewed 39 studies concerning parent–child agreement in health-related assessments to reveal overall agreement, directions of agreement, and the factors that affect agreement in ratings. Overall, parents are better (but not the best) at reporting objective information rather than subjective information about their child’s feelings and experiences [9]. However, there are still gaps in understanding how much children and their parents agree or disagree on a range of health-related assessments. It is crucial not only to have a good agreement between them but also between parents and clinicians. This helps to ensure that caregivers are fully informed about their children’s well-being and are actively involved in decisions related to their care. In fact, one report issued from the Department of Psychology, University of South Carolina, aimed to examine the association between clinician observation and parent report, not for general overall pediatric health, but for two key developmental domains at 9 and 12 months of age: social communication and motor skills [10]. Addressing overall pediatric health will reveal a better association between clinicians and parents.

The difference between parent-reported and clinician-observed measures of children’s general health has not been extensively studied. Inconsistencies have been found in previous research, highlighting the need for more investigation in this area in order to understand the level of agreement and the factors contributing to any observed discrepancies. This will help evaluate parents’ awareness of their children’s health. Also, while studies have suggested that parents may have poor knowledge of specific health issues or symptoms [11,12,13,14,15], there is a lack of research on parents’ overall awareness of their children’s full physical and mental health status. Parents’ awareness of bullying and vaccination [16,17] were extensively studied in Lebanon. However, without an overall awareness of kids’ health, it may be difficult to develop effective interventions aimed at improving parental knowledge and awareness of their children’s health. Therefore, identifying the domains with the highest mismatch between the medical and parental findings is crucial for targeted interventions and effective public health strategies. The inclusion of a diverse range of areas, including marginalized rural zones where a higher number of uneducated people reside, adds a robust dimension to the significance of the study in the Lebanese context. Therefore, this research appears as an important contribution, specifically to the Lebanese context and to socio-demographically weak groups such as rural communities, where dark health disparities exist.

This school health campaign was held by the Order of Malta Lebanon (OML) for children aged between 3 and 12 years all over the country. The OML is an apolitical humanitarian organization; the core of its many missions is the community health centers (OML medical centers and MMUs (Medical Mobile Units)) (http://orderofmaltalebanon.org/community-health-centers/ (accessed on 27 April 2023)) distributed all over Lebanon. This article presents a study that aims to address the above key gaps. To foster a more comprehensive and effective approach to pediatric healthcare in the Lebanese context, dealing with the key research gaps is essential. It will allow early detection and intervention that resonate with the individualized needs of each child. Perhaps parents are not aware of all the health issues affecting their children, but spotting these discrepancies can help with early intervention and prevention. Amid Lebanon’s economic crisis, especially over the past 3 years, more challenges are being confronted, including those related to healthcare infrastructure and access. The results we obtained may facilitate the allocation of resources in an efficient way and the development of targeted health education programs that would favor a better communication between parents and children, thus ensuring both parties have accurate information about specific health conditions. To investigate this issue, we compared parental reports (1) with clinical observations obtained through physician assessments, as well as (2) with children’s responses to a mental health interview conducted by nurses. We hypothesize that different degrees of mismatch concerning children’s health will be detected between medical assessments’ results and parents’ findings. Important insights into parental health literacy in Lebanon are discussed, offering the first comprehensive data on parents’ understanding and knowledge of their children’s overall health across multiple domains. The inclusion of a diverse range of areas, including marginalized rural zones where a higher number of uneducated people reside, adds a robust dimension to the significance of the study. The results of this research have the potential to inform the development and enhancement of health literacy programs, health information campaigns, and other social interventions aimed at parents and their school-aged children in Lebanon.

## 2. Materials and Methods

### 2.1. Study Design and Participants

**Figure 1:**
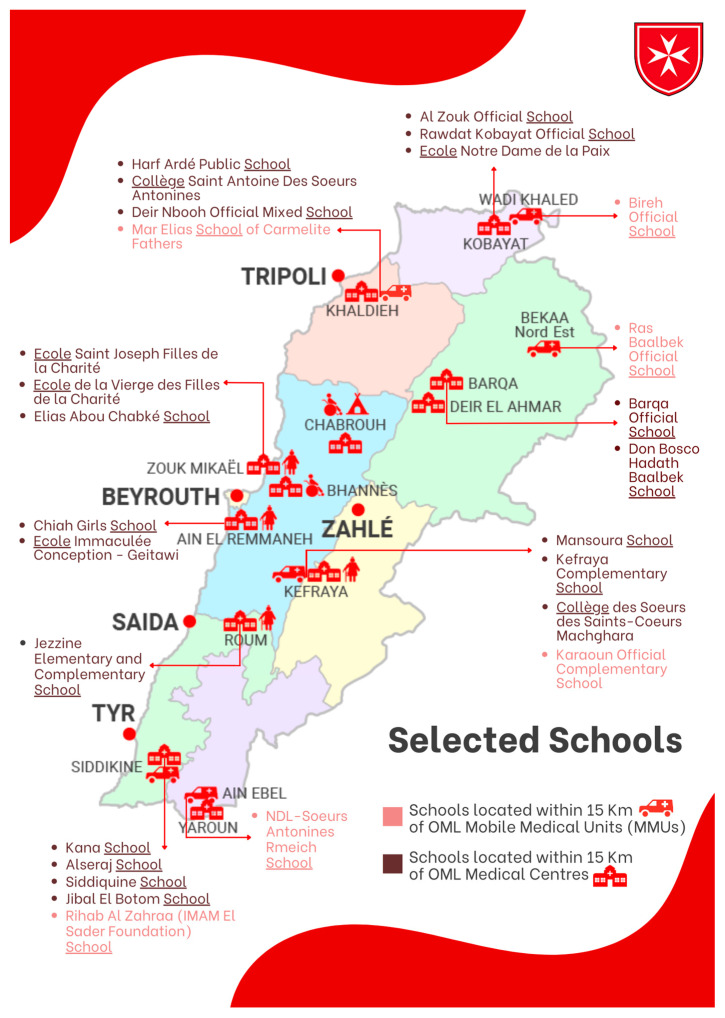
Selected schools in proximity of OML centers and MMUs. OML: Order of Malta Lebanon; MMUs: Mobile Medical Units.

The aim of this analytical study was to evaluate whether parents are aware of their children’s health by comparing parent’s answers with children’s medical evaluation and interviews performed by physicians and nurses. A large cross-sectional study was carried out in 27 schools widely distributed all over Lebanon, and in proximity with OML medical centers and MMUs (Medical Mobile Units). A total of 16 public schools and 11 semi-private ones with variable sizes were recruited in our project (Figure 1). We selected children of any nationality between 3 and 12 years of age (from kindergarten to Grade 7) who are in schools located at a maximum of 15 km from the OML medical centers and Mobile Medical Units (MMUs). For each center or MMU, around 2 to 3 public schools and 1 semi-private school were chosen. Excluded from the study were students in the mentioned grades who fell outside of the specified age range.

**Figure 2:**
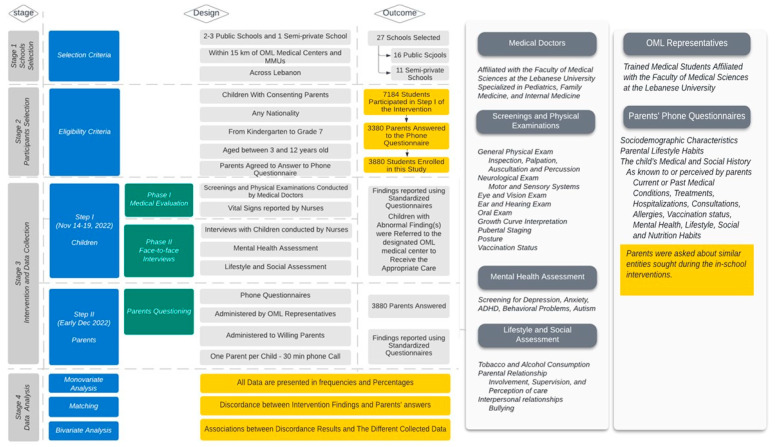
Study Flow. OML: Order of Malta; MMUs: Mobile Medical Units; Highlighted in yellow are the key elements directly related to the study objectives, serving as the main workflow of the research.

To realize the aims of this project, two important steps were conducted (Figure 2):

(1) The first step consists of the physical exam performed in schools by both a medical doctor and a nurse. Additionally, each child was interviewed in a private and suitable environment by the nurse to obtain information on their mental health. These interactions lasted approximately 10 to 15 min per student. This step occurred over the period of 14 November to 19 November 2022.
(2) The second step entailed interviewing the parents of these children. These interviews were conducted over the phone by very well-trained representatives of our organization, which consisted of medical students. After obtaining the parents’ consent, the parents were asked to respond to a questionnaire dealing with the physical and mental state of their children, modeling aspects that medical doctors and nurses examined during the first step. The aim of this step was to determine the differences between findings during medical visits and data provided by students to nurses compared to parents’ responses. Our analytical study emphasizes this discordance. The interviews with parents took place within the first ten days of December 2022 and lasted for about thirty to thirty-five minutes per phone call. In comparing the information collected from children and their parents, we aimed to determine the key gaps/discrepancies in this study or how they could shape individual perceptions of reported health conditions.

The majority of medical doctors were from the Faculty of Medical Sciences at the Lebanese University and specialized in Pediatrics, Family Medicine, or Internal Medicine. Lebanese university medical students were recruited as OML medical staff to interview children’s parents and complete the medical file of each child. They were trained to verify the parents’ consent, to deliver a message of introduction provided by the OML communication department, and to ensure the good quality of their performance.

### 2.2. Data Collection

Three separate questionnaires filled out over google form were used for data collection.

These questionnaires were carefully designed to address the unique aspects of our project in Lebanon and ensure a thorough examination of the research objectives. The first questionnaire, “Nurses’ findings”, reports vital signs and a face-to-face mental assessment interview with the child. This was performed and filled out by the nurses. The second questionnaire, “Doctors’ findings”, reports the screening and physical exam findings. This was filled out by the medical doctors during the auscultatory session (medical evaluation) of the child. The third questionnaire, “Parents’ questionnaire”, gathers the child’s medical history as well as their mental health. This form was filled out by the OML representatives over WhatsApp phone calls with the concerned parents.

The “Nurses’ findings” and “Doctors’ findings” parameters were prepared by the OML nurse and the OML pediatrician. These parameters were further detailed and developed by the first authors using several references [18,19,20]. The section about eating habits in the “Nurses’ findings” questionnaire was prepared by the OML nutritionist.

The “Parents’ questionnaire” was prepared according to a school health questionnaire by Dr Flore Martini [21] and by the OML nurse.

The child’s medical condition questions were specifically adapted, in a simpler way for parents and in a way that fits and matches with the doctors’ and nurses’ findings, in order to accurately compare parents’ answers with the medical visit findings.

It is essential to note that the structure of our questionnaires involved distinct sections for individual systems (nervous system, respiratory system, etc.). Each section forms a unit with questions relevant only to this particular aspect of health. This method guaranteed an in-depth analysis of every system together with relevant questions.

All three questionnaires were revised and edited by the research department. They were translated from English to French and Arabic languages using the inverted method of Fortin [22].

### 2.3. Variables

The data were collected from the “Nurses’ findings” questionnaire, the “Doctor’s findings” questionnaire, and from the parents’ questionnaire.

In this comparative analysis, we intentionally studied variables that were present in both the physician’s assessment and the parents’ questionnaire to allow for a direct and accurate comparison of responses between both parties: (1) the overlapping variables between the physician’s assessment and the parent’s answers. These variables are the following: vital signs, general physical exam, neurological exam, motor and sensory system, eye and ear exam findings, oral exam findings, pubertal stage, posture, and vaccination status; (2) and the overlapping variables between the face-to-face interview and the parents’ questionnaire answers. These variables are the following: mental assessment (Depression, ADHD, behavioral problems, worry and anxiety, temper tantrums, autism) including the parents’ supervision and care, relational and emotional life (bullying), and tobacco and alcohol consumption.

The selection of variables in this study was guided by our intention to comprehensively address various health aspects, spanning across every human system. We aimed to cover a broad spectrum of factors to ensure a good examination of mismatches.

### 2.4. Statistical Analysis

Data were analyzed using SPSS version 25.

Univariate analysis was chosen and all the study variables were presented. Nominal data (qualitative) were represented as frequencies and proportions. As for the continuous variables, results were presented by frequency means, standard deviations, and minimum and maximum values.

Matching: A matching methodology was used to assess the matched results between doctors’ questionnaire (reported by the physicians’ exam)/nurses’ questionnaire (reported by nurses in the face-to-face interview with the students) vs. parents’ findings (reported by the OML representatives’ phone interviews). Matched data were coded by “1” and unmatched data were coded by “0”, e.g., to assess the exposure of students to passive smoking, the dual-method approach helped us gather information from both students (by the nurses’ interview) and parents (phone call interview) and specific questions were tailored to capture the perspectives of both groups. Students were asked, “Were you subject to passive smoking in the last 7 days?”. Simultaneously, parents were queried with, “Does someone at home smoke in the presence of your child?”.

The following data were presented according to frequencies and proportions:

Physical assessment: Matching between Physician’s assessment and the parents’ answers: Matching Vital Signs (Temperature, Heart rate), Matching abnormal vision, Matching mental health problem (Depression, Behavioral Problems), Matching Infectious skin lesions (Mycosis, Scabies, Impetigo, Lice, Varicella, Measles), Matching Hypercholesterolemia (Eyelids, Elbows, Ankles, Fingers, Wrists, Corneal Arc), Matching abnormal lung auscultation, Matching abnormal thyroid (Hypothyroidism, Goiter), Matching enlarged node or/and organ, Matching ear abnormality (Recurrent Ear Infection, Ear Disorder), Matching Oral (Cavities, Aphthous ulcers, Tonsils’s enlarged), Matching early or late pubertal stage, and Matching Level of consciousness (attention, alertness, orientation, etc.). Matching Motor system: Weakness, Paralysis, Unsteady gait), Matching Skin color (Pale, Icteric, Cyanotic), Matching Eye exam (Strabismus), Matching Posture (Scoliosis), Matching Heart auscultation (Murmur), and Matching Vaccination (Updated).

Mental assessment and Lifestyle habits: Matching between nurses’ assessment (according to the face-to-face interview with the child) and the parents’ questionnaire answers: Matching BMI (Nutrition issues) and Matching Psychiatric problems (Behavioral Problem, Child withdrawal or loneliness, Worry and anxiety, Anxiety, Depression, Sadness, Bullying).

Categorization of mismatch was computed as shown in Table 1 (percent disagreement), from very extreme (>50%), to extreme (30–50%), to moderate (15–30%), and to mild (<15%) mismatch.

Matching was represented as a function of all the study variables (demographics of children, demographics of parents, school-related characteristics, and other study variables). The tests used in the bivariate analysis were the Chi-square test, Fisher exact test, independent t-test, and ANOVA test.

A statistically significant association was set at 5% (*p* value less than 0.05).

When assessing agreement/mismatch, the best advice for researchers is to calculate both the percent agreement and the kappa [23].

Therefore, each question was answered in Boolean form (normal/abnormal), allowing us to estimate the agreement between medical findings’ and parents’ answers using Cohen’s kappa.

Cohen’s kappa, symbolized by the lowercase Greek letter, κ, is a robust statistic intended to measure the agreement between two variables. Similar to correlation coefficients, it can range from −1 to +1, where 0 represents no agreement (complete mismatch of answers), and 1 represents perfect agreement (no mismatch) between the raters.

For each kappa coefficient, its standard error was calculated and a 95% confidence interval was constructed using a normal approximation [24].

Cohen suggested the kappa result be interpreted as follows: values ≤0 indicate no agreement and 0.01–0.20 none to slight, 0.21–0.40 fair, 0.41–0.60 moderate, 0.61–0.80 substantial, and 0.81–1.00 almost perfect agreement [23].

### 2.5. Ethical Consideration

Before we started our study, under the supervision of the Order of Malta Lebanon, we obtained approval from the Ministries of Public Health (MoPH)/Education (Approval number 3/10460 received on 25 October 2022). This study was conducted in accordance with Good Clinical Practice ICH Section three, and the principles laid down by the 18th World Medical Assembly (Helsinki, 1964) [25] and all applicable amendments. Responses were confidential and were only used for research purposes. Parents and their children were asked by the school administration to electronically sign an informed consent in Arabic if they agreed to participate voluntarily in our study. In the informed consent, a detailed explanation of the background, objectives, risks, and advantages of the study was provided.

## 3. Results

Our research project involved conducting medical screenings of 7184 students, with 3880 parents participating in interviews about their children’s physical and mental health. The findings of these evaluations were provided in the first publication of this project. The objective of the subsequent analysis is to identify any areas of disagreement/mismatch and the implicated factors by focusing on the parameters that the medical assessments and parent interviews have in common.

### 3.1. Socio-Demographic Characteristics

The socio-demographic data of the children whose parents accepted to be interviewed (N = 3380) are listed in Table 2. The total might have differed from one question to another since some questions were not obligatory and the number of family members (e.g., siblings or grand-parents or aunts living in the same house) varied (the total for each factor is also listed). Most children (65.8%) were in private schools and the remaining (34.2%) were in public ones. Their age range was between 3 and 12 years of age, the largest proportion being between 3 and 6 years (39.8%). The sample is quite evenly split between female (51.5%) and male students (48.5%). Regarding their health status, a considerable proportion of the pupils were underweight (42.5%), while 38.2% were overweight. In addition, 57.5% of the students had no medical history, but 42.4% had been hospitalized at least once. In terms of governorate, the highest number of students of the interviewed parents in our sample lived in the south governorate (27.4%), while the lowest number lived in Beirut (5.5%). The parents’ socio-economic status varied widely, with most families earning less than USD 100 per month (41.1%). Most mothers were housewives (70.3%) and a significant proportion of fathers were employed (43.4%). The interviewed population, while not a representative of the total number participating students (N = 7184), closely mirrors the population of school-aged children and their parents in Lebanon.

### 3.2. Mismatching and Their Socio-Demographic Factors

After comparing the overlapping data between the medical assessments and parent interviews, mismatches in some areas were found. Different levels of disagreement were assigned depending on the percentage of mismatch (Table 1). Table 3, Table 4, Table 5 and Table 6 compare the physician’s report/nurse’s interview with the students vs. the parent’s questionnaire regarding several health parameters related to the child’s physical and mental health. The level of disagreement between the two parties is indicated by the mismatch percentage.

**Figure 3:**
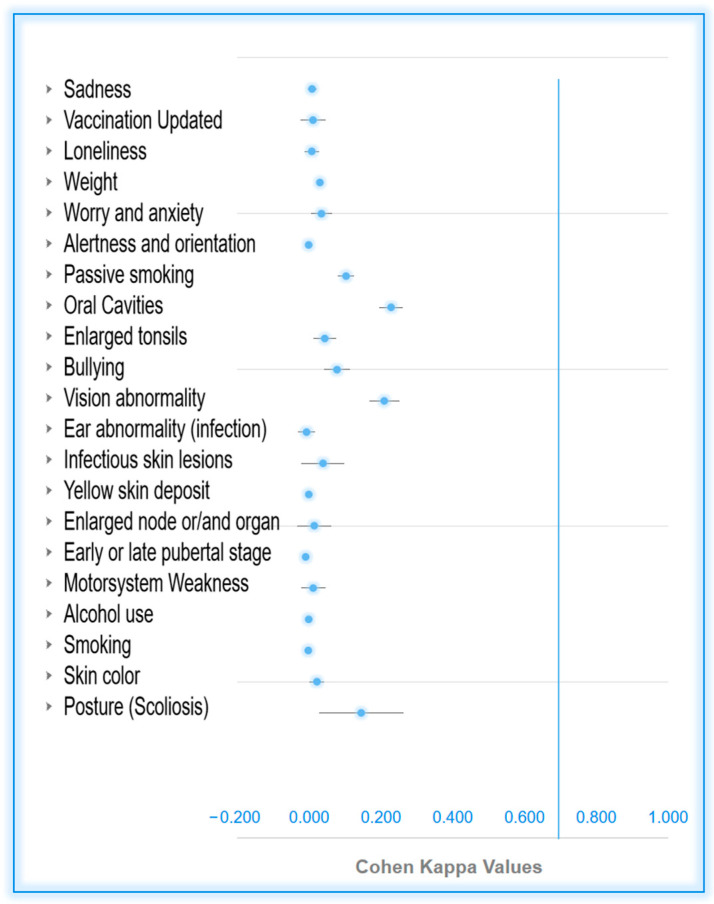
Forest plot displaying the Cohen’s kappa values. Vertical lines identify the items under investigation.

Table 3, Table 4, Table 5 and Table 6 also present the kappa values for the items under investigation, along with the corresponding forest plot below for visual representation (Figure 3).

It can be observed that items categorized as very extremely and extremely mismatched exhibited low kappa values, falling within the range of 0 to 0.2, signifying no to slight agreement.

However, the items of moderate and mild mismatch (better agreement) also had low kappa values falling within the same range. The occurrence of low kappa values for high agreement is linked to the elevated value of the expected proportion (Pe). The latter, used in the kappa formula *, represents the proportion of agreement by chance. The greater the expected chance of agreement, the lower the resulting value of the kappa [22]).

* The kappa formula is the observed probability (Po) of agreement minus the expected proportion (Pe) of agreement, divided by one minus the Pe.

In the subsequent sections, we outline the bivariate analysis conducted to highlight the associations between very extremely and extremely mismatched items and the implicated factors. For a more in-depth exploration of factors that were significantly correlated with these mismatches, additional detailed information is available in the Supplementary Tables S1 and S2.

### 3.3. Category of Items of Very Extreme Mismatch and Their Socio-Demographic Determinants

#### 3.3.1. Sadness

There was a 69.1% disagreement or discordance related to expressing sadness between children’s vs. parents’ answer (Table 3). Concerning its correlation with socio-demographic factors, the mismatch was found to be higher among students attending public schools (*p* = 0.0130) and residing in the Southern region of the country (77.2%), as opposed to those in the capital city Beirut (54.5%) (*p* < 0.001), and among children aged between 3 and 6 years old (*p* < 0.001). Moreover, the disagreement related to expressing sadness was found to be higher among single mothers (*p* = 0.036), and if the child often visited the pediatrician and has ongoing medical treatments (Table S1).

#### 3.3.2. Incomplete Vaccination

Similarly, for incomplete vaccination, there was a discordance of 67.8% between the doctor’s finding vs. parents’ answer about whether their child had an updated vaccination (Table 3). Factors that were found to be associated with a higher mismatch were children aged between 3- to 9-year-old, in private schools, and in Mount Lebanon. Also, families earning >USD 900 had the higher mismatch. The mother’s and father’s occupation (*p* < 0.001 and *p* = 0.004, respectively) and their level of education (*p* < 0.001) were also strongly correlated with the incomplete vaccination mismatch. Employed parents and those with a higher level of education achieved a higher mismatch than those with no education (Table S1).

#### 3.3.3. Withdrawal/Loneliness

A very extreme mismatch was also reported between the children’s answers (as reported to the nurse) and parents’ answers for the item of withdrawal or loneliness (67.8%) (Table 3). Several factors significantly influenced this mismatch such as the governorate, age group (3–6 years) (both *p* < 0.001), gender (with girls exhibiting a higher level of mismatch, *p* = 0.048), and mother’s occupation (specifically, children with working mothers, *p* = 0.041). Furthermore, children who visited a pediatrician were also more likely to experience withdrawal mismatch (Table S1).

#### 3.3.4. BMI

There was 59% discordance between the doctors’ findings and the parents’ answers regarding their children’s BMI, particularly whether the child had nutrition problems (Table 3). The factors that favor this discordance were for the child to be in a public school, within the older age group (10–12 years), and overweight (all *p* < 0.001), as well as the mother’s level of education (*p* = 0.024) and the child’s past medical history (*p* = 0.015) (Table S1).

#### 3.3.5. Anxiety/Worry

A very extreme mismatch was also reported between children’s and parents’ answers for the item of anxiety (53.4%) (Table 3). Implicated factors included living in the north governorate, being aged between 10 and 12 years, and having parents with a middle-income wage and who were not single or separated. Additionally, visiting a psychologist was also found to be a significant predictor of anxiety mismatch in children (Table S1).

#### 3.3.6. Level of Consciousness

There was a 52.5% discordance between doctors’ findings and the parents’ answers regarding their children’s level of consciousness (Table 3). All the sociodemographic determinants were associated with a mismatch in level of consciousness (*p* < 0.05), except for BMI, gender, the father’s level of education, and if the child had visits to the pediatrician/psychologist (Table S1).

### 3.4. Category of Items of Extreme Mismatch and Their Socio-Demographic Determinants

#### 3.4.1. Oral Cavities/Enlarged Tonsils

Between doctors’ findings and the parents’ answer, there was a 40.3% mismatch for the oral cavities and a 39.9% mismatch for the enlarged tonsils (Table 4). Both mismatches were significantly associated with children from public schools (*p* < 0.001), with the age of the children, with the mother’s occupation (with a lower mismatch percentage being attributed to mothers working in the health field), as well as with the parents’ level of education (*p* < 0.001) and marital status. Furthermore, there were mismatches regarding oral cavities in families with low to no income (*p* < 0.01) (Table S2).

#### 3.4.2. Bullying

It is known that parents may not talk openly with their children about their experiences with bullying. In this study, answers regarding bullying provided by the child (as reported to the nurse) differ by about 33% with parents’ answers (Table 4), and this was mainly seen in private schools (*p* < 0.001), in the Baalbek-Hermel governorate (*p* < 0.001), in families with a high monthly income (*p* = 0.039), in children in the older age group (10–12 years old, *p* < 0.001), and with those who go to a pediatrician/therapist (*p* < 0.05) (Table S2).

### 3.5. Category of Items of Moderate and Mild Mismatches

The items of moderate and mild mismatches are listed in Table 5 and Table 6, respectively.

In line with earlier research, we found more physician vs. parent and child vs. parent agreements on observable or external symptoms and behaviors than on non-observable domains, such as feelings and emotions. No correlations were established due to their high matching percentage; however, it is believed that a deeper understanding of the exact factors affecting these small discrepancies could help in improving them.

The items of moderate mismatch between doctor’s findings vs. parents’ answers were about a vision problem that had already been detected (20.5%) and ear infections/problems (18%) (Table 5).

The items of mild mismatch between doctor’s findings vs. parents’ answers were about infectious skin lesions, yellowish deposits on the skin, and the motor system, as listed in Table 6.

## 4. Discussion

**Figure 4:**
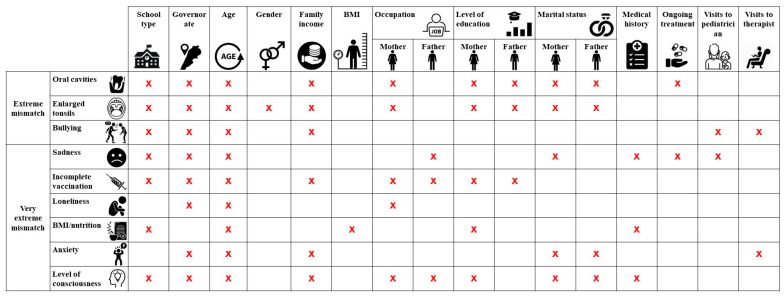
Factors significantly affecting mismatches between medical visit’s findings and parents’ answers. BMI: Body Mass Index. “x”: Statistically significant difference found at 5%.

Figure 4 summarizes the factors that affect entities of extreme and very extreme mismatch.

This study investigated the mismatch (discordance of answers), on the one hand, between physician’s vs. parents’ reports and, on the other hand, between children’s reports (as recorded during nurse’s interview) vs. parents’ reports, regarding various parameters in the domains of mental/physical health and behaviors. Overall, the results demonstrate more mismatch in non-observable domains (like mental health, such as feelings of anxiety and emotions (sadness)) than on observable or external symptoms (skin lesions, body posture, etc.). Parents seemed to report more accurately what they see rather than what the child feels or experiences. In line with earlier research, several studies found more agreement in observable/external symptoms and domains than in non-observable/internal ones, as stated by the systematic review article under the name “Agreements and disagreements between children and their parents in health-related assessments” [9]. This is actually extensively interpreted and explained throughout the discussion, but mismatching is mainly due to a lack of awareness and communication.

As evidenced by research in the literature [26], kappa values tend to be less reliable when dealing with infrequent observations. This could elucidate the observed low kappa values for items categorized as mild and moderate mismatch, despite having high agreement. This discrepancy may be attributed to the low prevalence of certain conditions, for instance, infectious skin lesions were only reported in 1.6% of children and posture problems in 2.2% of children (as reported in the first article of our research project by Habchy et al. [27]). Due to the limited occurrence of these items, the reliability of the kappa in accurately reflecting agreement is reduced, highlighting the significance of taking prevalence into account when interpreting kappa values. Our study is a screening study rather than an evaluation of a certain disease, therefore the kappa is not that reliable due to the low prevalence found concerning some diseases.

However, for items categorized as very extreme and extreme mismatch, the observed very low kappa values align with our expectations, as these low values signify “no” to “slight” agreement, with the latter indicating and supporting the high mismatch percentages. The reliability of the kappa in this context is supported by the presence of these diseases or concerns in a fair percentage within our population [27]. For example, consider the item “sadness and stress”, which is classified as very extreme mismatch. Its observed low kappa value of 0.008 aligns with our low agreement percentage, underscoring the true presence of disagreement. This result confirms the accuracy of our findings’, as the low kappa accurately reflects the actual discordance between medical findings and parental reports. Importantly, its fair prevalence of 26% [27] in our population supports the reliability of using the kappa in this context.

### 4.1. Physical Health

Observable or external symptoms organized by descending order of mismatch: Only a few items were categorized as very extremely and extremely mismatched according to doctors’ findings versus parents’ answers.

#### 4.1.1. Incomplete Vaccination

Misconceptions, false claims, and a lack of knowledge regarding vaccines significantly impact parents’ decisions to vaccinate their children. A study in Lebanon found that parents’ knowledge regarding childhood vaccination was moderate, with a mean knowledge score of 59.3% [17]. Our results showed that a higher mismatch for incomplete vaccination was observed in children from private schools. Actually, parents may assume private schools have robust health services that keep track of their children’s vaccination status, therefore, parents who can afford to send their children to private schools may be less inclined to prioritize vaccinations. In addition, the study cited above showed that a higher monthly income was associated with less knowledge and a negative attitude towards vaccination [17], which may explain the higher mismatch found in families earning over USD 900 per month. Conversely, in other studies, low income was shown to be associated with a negative attitude, as some parents may choose to spend money on other necessities [28]. This may be viewed as a social desirability bias, as some parents may have responded in a manner that is viewed as socially acceptable, such as indicating that they vaccinate their children, even if they do not. These reasons could explain the very extreme mismatch seen in our study regarding vaccination. Employed parents and those with higher education levels had a higher level of mismatch than those without education for the same reasons mentioned above. Along with other studies [29], this mismatch is even more pronounced in parents with younger children, since they are less likely to know the recommended vaccination schedule and the age their child must have started immunization. Some parents may not tell healthcare workers about their children’s vaccination status due to personal beliefs [30].

#### 4.1.2. BMI

Due to social norms and perception biases regarding body size, parents may perceive their child’s weight differently from how a healthcare professional would [31]. Additionally, they may not be aware of what constitutes a healthy weight for their child. Our study showed that the mismatch about BMI was very extreme and this mismatch between doctor’s findings and parents’ answer was significantly correlated with parents whose kids are overweight. A research study conducted in Lebanon [32] showed that overweight children’s parents are less aware and less knowledgeable of their child’s weight status and that parent–physician communication has a significant impact on parents’ knowledge. In addition, according to a study that appeared in the International Journal of Pediatric Obesity, parents of obese kids were more likely to think that their child’s weight was “normal” if the kid had a higher body mass index (BMI) percentile [12]. Our results also showed that children being in the older age group (10–12 years old) significantly affected the mismatch. There has not been enough research to know the reason behind this. However, one would think that, as they grow, children become more independent and spend less time with their parents, making it more challenging for them to keep track of their offspring’s weight. Conversely, other studies have revealed that parental awareness of their child’s weight condition is better with younger children [13]. Importantly, our results suggest that parents whose children attended public schools were less aware of their children’s weight than those who attended private ones. This is in line with other studies [33], and this may be due to the greater access to health in private schools. In the literature, some studies [4] showed that parents’ level of education and socioeconomic status affected the perception of weight, and our results showed a positive correlation with the mother’s level of education (less mismatch with educated mothers). Further studies are required to investigate the precise factors that might be responsible for variations in parental knowledge of their child’s weight status in various school settings.

#### 4.1.3. Oral Cavities/Enlarged Tonsils

Parents with lower incomes, who have children in public schools, and children in the lower age group had a higher level of mismatch regarding these two items. These parents may not prioritize their children’s oral health due to financial or insurance limitations. In addition, public schools may lack regular health checks, leading to a lack of parental awareness. There is insufficient evidence for the efficacy of primary school-based behavioral interventions for reducing caries [34]. Let us not forget to mention that little children might not visit the dentist as regularly as older children, which means that dental practitioners have fewer opportunities to inform parents about their children’s oral health conditions. Also, a higher mismatch was attributed to single mothers and fathers who face unique challenges in monitoring their children’s health, whereas a lower mismatch percentage was attributed to mothers working in the health field, who are more aware of their children’s health.

#### 4.1.4. Other Items (Vision Problems, Ear Problems, Skin Lesions, Apparent Skin Infection, Bad Posture, etc.)

Although these items are of moderate and mild mismatch, one should pay attention to the discordance regarding infectious lesions (scabies); parents might either lack the knowledge to identify scabies or lice or deny the condition, since their kid must interrupt classes if the teacher notices that the kid is infected, or simply due to financial or insurance limitations. This discord should be addressed through awareness campaigns to increase parents’ knowledge and promote better prevention.

### 4.2. Mental Health

Non-observable symptoms organized by descending order of mismatching: Multiple items were categorized as very extremely and extremely discordant according to doctors’ findings/interviews versus parents’ answers.

#### 4.2.1. Sadness

The mismatch between children’s and parents’ responses to sadness was very extreme, especially in public schools and southern regions. This may be due to differences in communication and parental involvement between public and private schools. Indeed, private schools offer better opportunities for parents to learn about their child’s emotional well-being.

Additionally, the mismatch about sadness was associated with younger children. Actually, according to a study analyzing the concordance between mothers’ reports and children’s self-reports of depressive symptoms, adult–child agreement improved as children became older [35]. Thus, younger children may be less able to express their emotions effectively and, thus, parental awareness of children’s sadness and loneliness increases with age. In addition, the mismatch was higher in single mothers. Being a single parent is a definite factor that increases the risk of mental health issues for both adults and children. In line with our results, another study found that “single parenthood raises further economic challenges compounding the level of stress, possibly causing more difficulties in parent–child relationships” [36] and, therefore, there is a lack of communication, explaining the lack parental awareness regarding their children’s signs of sadness. Furthermore, rural parents tend to avoid seeking mental health services for their children, possibly due to the stigma surrounding mental health in these areas, therefore leading to less awareness of their children’s signs of sadness [14,37].

#### 4.2.2. Withdrawal/Loneliness

Along with the other mental health mismatches, gender had an impact on the disagreement regarding withdrawal and loneliness between the children’s answer (during face-to-face nurses’ interview) and parents’ answers. Parents may have gender stereotypes and biases that could affect their perceptions of their kids’ well-being. Parents may perceive girls as being more social and boys as being more vulnerable to social isolation, so parents may overlook signs of loneliness in their daughters. Accordingly, a study showed that parents use more emotion words when talking with daughters than with sons [38]. Also, one study found that parents discuss loneliness and overall emotions more with their daughters rather than with their sons, and encourage boys to limit their emotions, which leads to less emotional awareness, including loneliness, in boys [39]. Not only was the gender parameter significant, but also ‘working mothers’, since they have less time to spend with their kids, and less interactions will make it more challenging for them to pick up on signs of loneliness [40]. The father’s occupation was not significant; this leads us to state that society places a greater pressure on mothers to balance work and family responsibilities.

#### 4.2.3. Anxiety and Bullying

Consistent with previous studies [15,41], the mismatch regarding anxiety between the children’s answer (during face-to-face nurses’ interview) and parents’ answers was very extreme. Salbach-Andrae et al. reported higher levels of disagreement for children who had internal mental health issues, such as anxiety [15]. As for bullying, parents are essential in recognizing and combating bullying behavior, but research indicates that many parents may not be aware that their children are being bullied [42]. In our findings, the mismatch for bullying was higher in private schools. We ask ourselves: Why are some parents in the dark in private schools? A reason for this could be the lack of transparency these schools have when it comes to reporting incidents of bullying. “We have unrealistic expectations of the virtues of a private education” stated Professor von Stumm in his study, “Bullying worse in private schools” [43]. Also, there is the perception of reputation; these parents may think that bullying is less of a problem in these schools because of their high academic standards, therefore this will lead to them being less vigilant about this topic. However, this contradicts the results of the mentioned Lebanese project about bullying, “The most aware mothers were some Lebanese mothers with children in private schools” [16].

The mismatches related to anxiety and bullying were also higher in rural areas (north and Baalbek governorates) due to the same reasons as for the sadness mismatch (that is, the stigma about mental health). ‘Save the Children’ is an NGO in Lebanon that commissioned a national study to investigate bullying among Lebanese, Syrian, and Palestinian children [16]. They found that the parents least aware of the concept were from the Beqaa Valley. Also, previous research has addressed how rural parents face social isolation and economic difficulties [44], which may lead to parents’ lower awareness of their children’s emotional well-being.

Unlike expressions of sadness and loneliness, anxiety and bullying’s mismatches were higher in the older age group (10–12 years old). As children become older, they may disclose less information to their parents about their anxiety and the bullying they experience and will develop coping mechanisms to deal with it on their own. Also, parents may be less likely to monitor their children like they used to when they were younger. Conversely, other studies found that parental awareness of anxiety symptoms and other mental health disorders did not significantly differ based on the child’s age [45,46]. We found a stronger mismatch for anxiety and also bullying in children who visit therapists. This might be the case because parents may rely too heavily on the therapist and consider the issue solved—“Half of the parents who had sought professional help tended to believe in improvement in their child’s anxiety” [47]—and, therefore, consider it gone. Also, therapy sessions give kids a place to safely express their feelings and deal with their anxieties, which may not be apparent to parents outside of therapy. Mixed and ambiguous results could be found in the literature regarding this matter due to the different types and durations of therapy.

### 4.3. Strengths and Limitations

This study is a cross-sectional study, and, as we know, it examines the association between exposure and outcome at a single point in time, and therefore cannot capture changes in exposure or outcome over time. To overcome this weakness as much as possible, the questions in the parents’ and children’s interviews were asked in a manner that covered a certain period of time (e.g., During the past 12 months, how often have you felt lonely?). The strengths of the study are the sample size and the range and variety of different behaviors and health outcomes investigated, from mental to physical health. However, some limitations should be acknowledged. To begin with, the social desirability bias certainly affected parents and children’s answers. The innate human tendency to present oneself in a socially desirable light may have influenced the responses from both parents and children. Such a bias may result in the overreporting of socially acceptable behaviors or positive health conditions while minimizing the disclosure for less socially desirable information. In our study, parents were asked about their child’s exposure to passive smoking. People might lean towards giving answers that fit in with what society sees as acceptable, especially when it comes to acknowledging the harm of smoking indoors. In addition, when it comes to the perception of harmlessness, this could lead holding back in terms of sharing certain details. People might skip over information they find sensitive or stigmatizing, like, for instance, when answering questions related to mental health. In addition, the information gathered from children and parents relies on self-reporting, which is susceptible to recall bias. Participants may not accurately remember or report certain details, affecting the precision of our findings.

Moreover, the study was unable to identify whether parent or child reports are more accurate, since we lacked a reliable indicator of the kids’ actual, “genuine” behavior and children seem to base their responses on a single example. So, a percentage of the mismatch seen in mental health questions could be due to the different reasonings, interpretations, and response styles between children and parents; the latter might be the ones with the correct answers. In addition, sometimes parents have the potential to over-report emotional and behavioral problems in their kids, as well as to see their child’s behavior as more problematic than the children themselves. Furthermore, reporting bias (under and overreporting biases) are present from both parties (children and parents) either to hide reality (stigma, judgments) or to grab attention (to obtain financial aid, for mental health help, etc.). We believe that children’s views and inputs are important, but a multi-informant approach is needed to exactly understand the situation.

Regarding the statistical methods, the kappa value and percent agreement have strengths and limitations. One major flaw in the percent agreement is that it does not account for the likelihood that raters guessed on scores. It might exaggerate the actual degree of agreement. In addition, the kappa was created to account for the risk of guessing and may inadvertently lower the estimate of agreement [23] and, most importantly, may not be a reliable coefficient if the disease prevalence is low [26] (as shown in our study).

### 4.4. Perspectives

All referrals have been presently addressed to the OML centers; therefore, every child is currently benefiting from a medical follow-up to the nearest OML medical center! Additionally, all participating children were further registered in the OML patients’ database to benefit from the various medical services provided by health centers. Most importantly, the medical team communicated with the school administration, which informed children’s parents about health findings of which they were not aware, thus favoring a better communication between parents and their children.

## 5. Conclusions

Critical mismatching has been identified between medical visit findings versus parents’ answers. A significant number of parents were unaware of their children’s physical and mental health status. Good communication between parents and pediatricians or physicians is needed to provide parents with better knowledge regarding weight, vaccination, and other health-related problems. In addition, it is important for working parents to make time for their children and maintain open lines of communication to better understand their children’s emotional states. Through educational campaigns, parents’ knowledge of the warning signs and symptoms of mental health problems in their kids can be increased, as well as the idea of getting professional help when necessary. Also, parents should monitor their children’s oral health, even if they attend private schools. They should schedule routine dental exams, keep an eye on brushing routines, and be alert to any potential dental problems. Furthermore, it is necessary to develop oral health education initiatives for parents to enhance their understanding of their children’s dental health. In conclusion, our findings revealed significant differences between medical diagnosis and parental reports; this is a promising zone for future research. Researchers can further investigate the exact underlying causes of each mismatch. Knowing what exactly caused these can guide the postulation of such interventions aimed at enhancing information accuracy. Understanding the discrepancies in health knowledge between children and parents can inform future targeted interventions to improve health literacy and ensure that both parents and children have accurate information about specific health conditions. Last but not least, our study showed the importance of schools in children’s health; more school-related health campaigns that involve parents are needed because these are important for promoting better health in children. Collaboration between parents and schools can be effective in promoting healthy behaviors, as well as making parents more aware of their kids’ behaviors. By working together, parents and schools can create environments that support healthy habits and help children develop lifelong healthy behaviors.

## Figures and Tables

**Table 1:** Classification of mismatch between intervention findings and parents’ answers.

	Mismatch Percentage
Very extreme	>50%
Extreme	30–50%
Moderate	15–30%
Mild	<15%

**Table 2:** Socio-demographic characteristics of participants.

			Frequency		Percentage	
Nationality Total = 3380	Lebanese Non-Lebanese	Syrian Palestinian Other	3232 148	137 3 8	95.6 4.4	4.1 0.1 0.2
Gender Total = 3380	Male Female		1640 1740		48.5 51.5	
Age Total = 3380	3–6 years 7–9 years 10–12 years		1344 1012 1024		39.8 29.9 30.3	
Body Mass Index (BMI) Total = 3380	Underweight Normal weight Overweight		1436 653 1291		42.5 19.3 38.2	
School type Total = 3380	Public Private		1156 2224		34.2 65.8	
OML community health facilities Total = 3380	Medical Mobile Units (MMUs) Medical centers		643 2737		19.0 81.0	
Governorate Total = 3380	Beirut Mount Lebanon North/Akkar Beqaa/Baalbek Hermel South		187 758 797 712 926		5.5 22.4 23.6 21.1 27.4	
Family Income Total = 2996	No income <USD 100 USD 100–300 USD 300–600 USD 600–900 >USD 900		277 1231 332 902 227 27		9.2 41.1 11.1 30.1 7.6 0.9	
Mother’s occupation Total = 3201	No work Housewife Student Unemployed Employed Self-employed Retired Disabled Health field Other		3 2251 16 88 589 134 9 2 76 33		0.1 70.3 0.5 2.7 18.4 4.2 0.3 0.1 2.4 1.0	
Father’s Occupation Total = 3093	No work Housewife Student Unemployed Employed Self-employed Retired Disabled Health field Other		8 5 11 213 1343 1110 118 17 35 233		0.3 0.2 0.4 6.9 43.4 35.9 3.8 0.5 1.1 7.5	
Mother’s level of education Total = 3242	No education Primary Complementary Secondary Undergraduate University graduate		73 399 722 705 436 907		2.3 12.3 22.3 21.7 13.4 28.0	
Father’s level of education Total = 3118	No education Primary Complementary Secondary Undergraduate University graduate		132 592 955 703 283 453		4.2 19.0 30.6 22.5 9.1 14.5	
Mother’s marital status Total = 3242	Single Married Divorced Widowed		97 3045 71 29		3.0 93.9 2.2 0.9	
Father’s marital status Total = 3118	Single Married Divorced Widowed		46 3031 36 5		1.5 97.2 1.2 0.2	
Mother’s smoking status Total = 3242	Never Former Occasional Current		1939 92 393 818		59.8 2.8 12.1 25.2	
Father’s smoking status Total = 3118	Never Former Occasional Current		986 170 296 1666		31.6 5.5 9.5 53.4	
Positive medical history Total = 2240	No Yes		1139 1101		50.8 49.2	
Positive surgical history Total = 3379	No Yes		2949 430		87.3 12.7	
Ongoing treatment Total = 1985	No Yes		1680 305		84.6 15.4	
Child visit to the pediatrician/doctor Total = 1969	No Yes		412 1557		20.9 79.1	
Previous child visit to the psychologist Total = 3339	No Yes		3196 143		95.7 4.3	
Parent belief in the importance of medical examination at school Total = 1970	No Yes Doesn’t know		56 1893 21		2.8 96.1 1.1	

**Table 3:** Very extremely mismatched items between intervention findings and parents’ answers.

Physician’s Report/Nurse’s Interview	Parents’ Questionnaire	Mismatch Percentage	Kappa Value
How often have you been sad or stressed?	Does your child express sadness?	69.1%	0.008
Referral for incomplete vaccination	Are your child’s vaccinations up to date?	67.8%	0.011
How often have you felt lonely?	Is your child very shy or even withdrawn?	61.0%	0.007
BMI (underweight, overweight)	Does your child have problems with nutrition? Do you think your child is of normal weight? Too skinny? Overweight/obese?	59.0%	0.03
How often have you been so worried about something that you could not sleep at night?	Is your child often anxious?	53.4%	0.034
Level of consciousness (attention, alertness, orientation…)	Does your child have now or recently had a problem of being alert and oriented?	52.5%	−0.001

**Table 4:** Extremely mismatched items between intervention findings and parents’ answers.

Physician’s Report/Nurse’s Interview	Parents’ Questionnaire	Mismatch Percentage	Kappa Value
Were you subject to passive smoking in the last 7 days?	Does someone at home smoke in the presence of your child?	47.3%	0.103
Oral examination—Cavities	Does your child have now or recently had cavities?	40.3%	0.228
Oral examination—Enlarged tonsils	Does your child have now or recently had enlarged tonsils	39.9%	0.044
During the past 30 days, how many days were you bullied?	Is your child bullied by people around them?	32.9%	0.078

**Table 5:** Moderately mismatched items between intervention findings and parents’ answers.

Physician’s Report/Nurse’s Interview	Parents’ Questionnaire	Mismatch Percentage	Kappa Value
Abnormal vision exam	Does your child have a vision problem that has already been detected?	20.5%	0.209
Abnormal ear exam	Has your child had or still has recurrent ear infections?	18.0%	−0.007

**Table 6:** Mildly mismatched items between intervention findings and parents’ answers.

Physician’s Report/Nurse’s Interview	Parents’ Questionnaire	Mismatch Percentage	Kappa Value
Infectious skin lesions (Mycosis, Impetigo, Scabies, etc.)	Does your child have any skin lesions?	3%	0.039
Yellowish deposits (hypercholesterolemia)	Does your child have any yellowish deposits on the skin?	0.8%	−0.001
Enlarged node or/and organ	Does your child have now or recently had persistent swollen glands or lymph nodes?	2.8%	0.014
Early or late pubertal stage	Has your child had or still have an early or late pubertal stage?	3.1%	−0.010
Motor system (Weakness, Paralysis, Unsteady Gait, etc.)	Does your child have now or recently had a weakness, paralysis, or an unsteady gait?	10.9%	0.011
During the past 30 days, how many days did you have at least one drink containing alcohol?	Does your child drink alcohol?	7.2%	−0.001
During the past 30 days, how many days did you use tobacco products?	Does your child smoke?	10.5%	−0.002
Skin color (Pale, Cyanotic, etc.)	Does your child have now or recently had an abnormal skin color?	5.2%	0.022
Posture (Lordosis, Kyphosis, Scoliosis)	Does your child have now or recently had a problem in his posture (scoliosis, etc.)?	2.5%	0.145

## Data Availability

The data presented in this study are available on request from the corresponding author. The data are not publicly available due to their containing information that could compromise the privacy of research participants.

## Supplementary Materials

The following supporting information can be downloaded at: https://www.mdpi.com/article/10.3390/children11020214/s1, Table S1. Sociodemographic and other factors affecting entities of very extreme mismatch. Table S2. Sociodemographic and other factors affecting entities of extreme mismatch.

## References

[1] Johnston R., Fowler C., Wilson V., Kelly M. (2015). Opportunities for Nurses to Increase Parental Health Literacy: A Discussion Paper. Issues Compr. Pediatr. Nurs..

[2] Morrison A.K., Glick A.F., Yin H.S. (2019). Health Literacy: Implications for Child Health. Pediatr. Rev..

[3] Sanders L., Shaw J.S., Guez G., Baur C., Rudd R.E. (2009). Health Literacy and Child Health Promotion: Implications for Research, Clinical Care, and Public Policy. Pediatrics.

[4] De Buhr E., Tannen A. (2020). Parental Health Literacy and Health Knowledge, Behaviours and Outcomes in Children: A Cross-Sectional Survey. BMC Public Health.

[5] Modern University of Business and Science Supporting School Health in Disadvantaged Rural Areas in Bekaa and Southern Lebanon. https://www.mubs.edu.lb/Uploads/who_report.pdf.

[6] Allensworth D., Lawson E., Nicholson L., Wyche J., Institute of Medicine (US) (1997). Committee on Comprehensive School Health Programs in Grades K-12. Schools & Health: Our Na-tion’s Investment Allensworth.

[7] Pomerantz E.M., Moorman E.A., Litwack S.D. (2007). The How, Whom, and Why of Parents’ Involvement in Children’s Academic Lives: More Is Not Always Better. Rev. Educ. Res..

[8] Martí M.A., Merz E.C., Repka K.R., Landers C., Noble K.G., Duch H. (2018). Parent Involvement in the Getting Ready for School Intervention Is Associated with Changes in School Readiness Skills. Front. Psychol..

[9] Hemmingsson H., Ólafsdóttir L.B., Egilson S.Þ. (2016). Agreements and Disagreements between Children and Their Parents in Health-Related Assessments. Disabil. Rehabil..

[10] Federico A., Shi D., Bradshaw J. (2021). Agreement between Parental Report and Clinician Observation of Infant Developmental Skills. Front. Psychol..

[11] Poulain T., Vogel M., Meigen C., Spielau U., Hiemisch A., Kieß W. (2020). Parent-Child Agreement in Different Domains of Child Behavior and Health. PLoS ONE.

[12] Killion L., Hughes S.O., Wendt J.C., Pease D., Nicklas T.A. (2006). Minority Mothers’ Perceptions of Children’s Body Size. Int. J. Pediatr. Obes..

[13] Ruiter E.L.M., Saat J.J.E.H., Molleman G., Fransen G.A.J., Van Der Velden K., Van Jaarsveld C.H.M., Engels R.C.M.E., Assendelft W.J.J. (2020). Parents’ Underestimation of Their Child’s Weight Status. Moderating Factors and Change over Time: A Cross-Sectional Study. PLoS ONE.

[14] Williams S.L., Polaha J. (2014). Rural Parents’ Perceived Stigma of Seeking Mental Health Services for Their Children: Development and Evaluation of a New Instrument. Psychol. Assess..

[15] Weems C.F., Feaster D.J., Horigian V.E., Robbins M.S. (2010). Parent and Child Agreement on Anxiety Disorder Symptoms Using the DISC Predictive Scales. Assessment.

[16] Save the Children. Bullying in Lebanon. Lebanon October 2018. https://lebanon.savethechildren.net/sites/lebanon.savethechildren.net/files/library/Bullying%20in%20Lebanon-%20Full%20research.pdf.

[17] Matta P., Mouallem R.E., Akel M., Hallit S., Khalife M.-C.F. (2020). Parents’ Knowledge, Attitude and Practice towards Children’s Vaccination in Lebanon: Role of the Parent-Physician Communication. BMC Public Health.

[18] Pediatric History & Physical Exam (Children Are Not Just Little Adults). https://www.ped.med.utah.edu/cai/howto/H&P%20write-up.pdf.

[19] Pediatric Health History Questionnaire. https://www.healthparkpediatrics.com/wp-content/uploads/2021/10/Initial-Health-Questionnaire.pdf.

[20] Initial History Questionnaire. https://morehousehealthcare.com/documents/pediatric-initial-history-questionnaire.pdf.

[21] Questionnaire Parental en vue de la Visite avec le Médecin de l’éducation Nationale. http://www.abdelmalek-sayad-nanterre.ac-versailles.fr/IMG/pdf/questionnaire_parent_visite_medicale.pdf.

[22] Fortin M.-F., Gagnon J. (2016). Fondements et Etapes du Processus de Recherche: Méthodes Quantitatives et Qualitatives.

[23] McHugh M.L. (2012). Interrater reliability: The kappa statistic. Biochem. Medica.

[24] Chmura Kraemer H., Periyakoil V.S., Noda A. (2002). Kappa coefficients in medical research. Stat. Med..

[25] World Medical Association (2013). World Medical Association Declaration of Helsinki: Ethical principles for medical research involving human subjects. JAMA.

[26] Viera A.J., Garrett J.M. (2005). Understanding interobserver agreement: The kappa statistic. Fam. Med..

[27] Habchy P., Tahan L., Moussi C., Barakat M.A., Ghanem L., Kattan O., Njeim A., Abou Habib L., El Bitar W., El Asmar B. (2024). Referrals and Determinant Factors of a National School Health Campaign in Lebanon on Children Aged between 3 and 12 Years Old. Children.

[28] Sakai Y. (2018). The Vaccination Kuznets Curve: Do Vaccination Rates Rise and Fall with Income?. J. Health Econ..

[29] Kanyi L. (2021). Factors Influencing Knowledge and Attitude of Mothers Towards Immunization of Children Under-Five Years in Farato, Gambia. Texila Int. J. Public Health.

[30] Becker M.H. (1974). The Health Belief Model and Sick Role Behavior. Health Educ. Monogr..

[31] Karimy M., Armoon B., Fayazi N., Koohestani H.R. (2019). A Study on the Knowledge, Attitude, and Practices of Iranian Mothers towards Childhood Obesity. Obes. Facts.

[32] Zoghby H.B., Sfeir E., Akel M., Malaeb D., Obeïd S., Hallit S. (2022). Knowledge, Attitude and Practice of Lebanese Parents towards Childhood Overweight/Obesity: The Role of Parent-Physician Communication. BMC Pediatr..

[33] Hood N., Colabianchi N., Terry-McElrath Y.M., O’Malley P.M., Johnston L.D. (2014). Physical Activity Breaks and Facilities in US Secondary Schools. J. Sch. Health.

[34] Cooper A.M., O’Malley L., Elison S., Armstrong R., Burnside G., Adair P., Dugdill L., Pine C.M. (2013). Primary School-Based Behavioural Interventions for Preventing Caries. Cochrane Database Syst. Rev..

[35] Renouf A.G., Kovács M. (1994). Concordance between Mothers’ Reports and Children’s Self-Reports of Depressive Symptoms: A Longitudinal Study. J. Am. Acad. Child Adolesc. Psychiatry.

[36] Behere A.P., Basnet P., Campbell P. (2017). Effects of Family Structure on Mental Health of Children: A Preliminary Study. Indian J. Psychol. Med..

[37] Van Vulpen K.S., Habegar A., Simmons T. (2018). Rural School-Based Mental Health Services: Parent Perceptions of Needs and Barriers. Child. Sch..

[38] Kuebli J., Fıvush R. (1992). Gender Differences in Parent-Child Conversations about Past Emotions. Sex Roles.

[39] Chaplin T.M., Aldao A. (2013). Gender Differences in Emotion Expression in Children: A Meta-Analytic Review. Psychol. Bull..

[40] Hossain M.I., Nasrin N., Halim S.F.B., Ahmed S. (2022). Parental Awareness towards Child Health: A Study on the Parents of High School Students at Khulna City Corporation in Bangladesh. Khulna Univ. Stud..

[41] Reuterskiöld L., Öst L., Ollendick T.H. (2008). Exploring Child and Parent Factors in the Diagnostic Agreement on the Anxiety Disorders Interview Schedule. J. Psychopathol. Behav. Assess..

[42] Matsunaga M. (2009). Parents Don’t (Always) Know Their Children Have Been Bullied: Child-Parent Discrepancy on Bullying and Family-Level Profile of Communication Standards. Hum. Commun. Res..

[43] Tes Magazine Bullying Worse in Private Schools, New Research Shows 2020. https://www.tes.com/magazine/archive/bullying-worse-private-schools-new-research-shows.

[44] Girio-Herrera E., Owens J.S., Langberg J.M. (2013). Perceived Barriers to Help-Seeking among Parents of At-Risk Kindergarteners in Rural Communities. J. Clin. Child Adolesc. Psychol..

[45] Carlston D., Ogles B.M. (2008). Age, Gender, and Ethnicity Effects on Parent–Child Discrepancy Using Identical Item Measures. J. Child Fam. Stud..

[46] Canavera K., Wilkins K.C., Pincus D.B., Ehrenreich–May J. (2009). Parent–Child Agreement in the Assessment of Obsessive-Compulsive Disorder. J. Clin. Child Adolesc. Psychol..

[47] Beato-Fernández L., Barros L., Pereira A.I. (2018). Father’s and Mother’s Beliefs about Children’s Anxiety. Child Care Health Dev..

