# Supplementary material for: A National School Health Campaign in Lebanon on Children Aged between 3 and 12 Years Old: Concordance Level between Parents’ Reports and Medical Visit Findings about Physical and Mental Health
Source: Children (Basel). 2024 Feb 7;11(2):214. doi: 10.3390/children11020214 (PMC10887313; doi:10.3390/children11020214)
Supplement: Supplementary file 1 [file children-11-00214-s001.zip › children-2827251-supplementary.pdf]

children

Article

A National School Health Campaign in Lebanon on Children Aged between 3 and 12 Years Old: Concordance Level between Parents' Reports & Medical Visit Findings about Physical & Mental Health

Léa Tahan [1,†], Peter Habchy [1,†], Charbel Moussi [1,†], Tia Khadra [1,‡], Melissa Jawich [1,‡], Alain Njeim [1,‡], Ogarite Kattan[1,‡], Leila Abou Habib [2], Wassim El Bitar [2,3], Béchara El Asmar [2,4,5] and Mirna N. Chahine[1,2,6,7*,§]

Supplementary materials

p* = p < 0.05

NS = Non-significant p value > 0.05

Table S1. Sociodemographic and other factors affecting entities of very extreme mismatch.

		Level of consciousness			Withdrawal/loneliness			Anxiety			Sadness			Incomplete Vaccination			BMI Nutrition		
		Mismatch	Match		Mismatch	Match		Mismatch	Match		Mismatch	Match		Mismatch	Match		Mismatch	Match	
**School type**	Public	525 (45.4)	631 (54.6)	*p**	718 (62.1)	438 (37.9)	*NS*	637 (55.1)	519 (44.9)	*NS*	830 (71.8)	326 (28.2)	*p**	698 (60.4)	458 (39.6)	*p**	711 (61.5)	445 (38.5)	*p**
	Private	1249 (56.2)	975 (43.8)		1345 (60.5)	879 (39.5)		1167 (52.5)	1057 (47.5)		1504 (67.6)	720 (32.4)		1593 (71.6)	631 (28.4)		1290 (58.0)	934 (42.0)	
**Governorate**	Beirut	77 (41.2)	110 (58.8)	*p**	119 (63.6)	68 (36.4)	*p**	83 (44.4)	104 (55.6)	*p**	102 (54.5)	85 (45.5)	*p**	127 (67.9)	60 (32.1)	*p**	119 (63.6)	68 (36.4)	*NS*
	Mount Lebanon	429 (56.6)	329 (43.4)		513 (67.7)	245 (32.3)		451 (59.5)	307 (40.5)		577 (76.1)	181 (23.9)		583 (76.9)	175 (23.1)		467 (61.6)	291 (38.4)	
	North - Akkar	370 (46.4)	427 (53.6)		472 (59.2)	325 (40.8)		486 (61.0)	311 (39.0)		554 (69.5)	243 (30.5)		540 (67.8)	257 (32.2)		464 (58.2)	333 (41.8)	
	Bekaa, Baalbek Hermel	313 (44.0)	399 (56.0)		387 (54.4)	325 (45.6)		380 (53.4)	332 (46.6)		386 (54.2)	326 (45.8)		412 (57.9)	300 (42.1)		419 (58.8)	293 (41.2)	
	South	585 (63.2)	341 (36.8)		572 (61.8)	354 (38.2)		404 (43.6)	522 (56.4)		715 (77.2)	211 (22.8)		629 (67.9)	297 (32.1)		532 (57.5)	394 (42.5)	
**Age**	3 - 6 years	592 (44.0)	752 (56.0)	*p**	879 (65.4)	465 (34.6)	*p**	694 (51.6)	650 (48.4)	*p**	1067 (79.4)	277 (20.6)	*p**	921 (68.5)	423 (31.5)	*p**	600 (44.6)	744 (55.4)	*p**
	7 - 9 years	587 (58)	425 (42)		595 (58.8)	417 (41.2)		542 (53.6)	470 (46.4)		654 (64.6)	358 (35.4)		693 (68.5)	319 (31.5)		675 (66.7)	337 (33.3)	
	10 - 12 years	595 (58.1)	429 (41.9)		589 (57.5)	435 (42.5)		568 (55.5)	456 (44.5)		613 (59.9)	411 (40.1)		677 (66.1)	347 (33.9)		726 (70.9)	298 (29.1)	
**Gender**	Male	853 (52)	787 (48)	*NS*	973 (59.3)	667 (40.7)	*NS*	848 (51.7)	792 (48.3)	*NS*	1157 (70.5)	483 (29.5)	*NS*	1101 (67.1)	539 (32.9)	*NS*	1243 (47.9)	397 (50.6)	*NS*
	Female	921 (52.9)	819 (47.1)		1090 (62.6)	650 (37.4)		956 (54.9)	784 (45.1)		1177 (67.6)	563 (32.4)		1190 (68.4)	550 (31.6)		1353 (52.1)	387 (49.4)	
**Family Income**	No income	156 (56.3)	121 (43.7)	*p**	157 (56.7)	120 (43.3)	*NS*	130 (46.9)	147 (53.1)	*p**	179 (64.6)	98 (35.4)	*NS*	175 (63.2)	102 (36.8)	*p**	160 (57.8)	117 (42.2)	*NS*
	<100$	617 (50.1)	614 (49.9)		738 (60.0)	493 (40.0)		690 (56.1)	541 (43.9)		806 (65.5)	425 (34.5)		773 (62.8)	458 (37.2)		743 (60.4)	488 (39.6)	
	100-300$	177 (53.3)	155 (46.7)		205 (61.7)	127 (38.3)		193 (58.1)	139 (41.9)		226 (68.1)	106 (31.9)		234 (70.5)	98 (29.5)		198 (59.6)	134 (40.4)	
	300-600$	516 (57.2)	386 (42.8)		557 (61.8)	345 (38.2)		443 (49.1)	459 (50.9)		617 (68.4)	285 (31.6)		627 (69.5)	275 (30.5)		534 (59.2)	368 (40.8)	
	600-900$	145 (63.9)	82 (36.1)		153 (67.4)	74 (32.6)		119 (52.4)	108 (47.6)		163 (71.8)	64 (28.2)		174 76.7)	53 (23.3)		145 (63.9)	82 (36.1)	
	>900$	15 (55.6)	12 (44.4)		13 (48.1)	14 (51.9)		12 (44.4)	15 (55.6)		17 (63.0)	10 (37.0)		20 (74.1)	7 (25.9)		14 (51.9)	13 (48.1)	
**BMI**	Underweight	641 (78.6)	175 (21.4)	*NS*	637 (78.1)	179 (21.9)	*NS*	541 (66.3)	275 (33.7)	*NS*	701 (85.9)	115 (14.1)	*NS*	578 (70.8)	238 (29.2)	*NS*	750 (52.2)	686 (47.8)	*p**
	Normal weight	1042 (77.6)	300 (22.4)		1092 (81.4)	250 (18.6)		883 (65.8)	459 (34.2)		1147 (85.5)	195 (14.5)		961 (71.6)	381 (28.4)		326 (49.9)	327 (50.1)	
	Overweight	855 (75.7)	274 (24.3)		927 (82.1)	202 (17.9)		763 (67.6)	366 (32.4)		941 (83.3)	188 (16.7)		803 (71.1)	326 (28.9)		925 (71.6)	366 (28.4)	
**Mother's occupation**	No Work	1 (33.3)	2 (66.7)	*p**	2 (66.7)	1 (33.3)	*p**	1 (33.3)	2 (66.7)	*NS*	3 (100.0)	0 (0.0)	*NS*	3 (100.0)	0 (0.0)	*p**	3 (100.0)	0 (0.0)	*NS*
	Housewife	1226 (54.5)	1025 (45.5)		1332 (59.2)	919 (40.8)		1178 (52.3)	1073 (47.7)		1509 (67.0)	742 (33.0)		1470 (65.3)	781 (34.7)		1343 (59.7)	908 (40.3)	
	Student	10 (62.5)	6 (37.5)		9 (56.3)	7 (43.8)		7 (43.8)	9 (56.3)		12 (75.0)	4 (25.0)		12 (75.0)	4 (25.0)		9 (56.3)	7 (43.8)	
	Employed	307 (52.1)	282 (47.9)		391 (66.4)	198 (33.6)		324 (55.0)	265 (45.0)		437 (74.2)	152 (25.8)		451 (76.6)	138 (23.4)		343 (58.2)	246 (41.8)	
	Self-employed	77 (57.5)	57 (42.5)		76 (56.7)	58 (43.3)		64 (47.8)	70 (52.2)		94 (70.1)	40 (29.9)		87 (64.9)	47 (35.1)		78 (58.2)	56 (41.8)	
	Unemployed	35 (39.8)	53 (60.2)		55 (62.5)	33 (37.5)		43 (48.9)	45 (51.1)		59 (67.0)	29 (33.0)		56 (63.6)	32 (36.4)		57 (64.8)	31 (35.2)	
	Retired	3 (33.3)	6 (66.7)		8 (88.9)	1 (11.1)		8 (88.9)	1 (11.1)		7 (77.8)	2 (22.2)		8 (88.9)	1 (11.1)		6 (66.7)	3 (33.3)	
	Disabled	0 (0.0)	2 (100.0)		0 (0.0)	2 (100.0)		1 (50.0)	1 (50.0)		1 (50.0)	1 (50.0)		2 (100.0)	0 (0.0)		1 (50.0)	1 (50.0)	
	Health field	40 (52.6)	36 (47.4)		46 (60.5)	30 (39.5)		44 (57.9)	32 (42.1)		50 (65.8)	26 (34.2)		53 (69.7)	23 (30.3)		38 (50.0)	38 (50.0)	
	Other	12 (36.4)	21 (63.6)		21 (63.6)	12 (36.4)		13 (39.4)	20 (60.6)		24 (72.7)	9 (27.3)		26 (78.8)	7 (21.2)		20 (60.6)	13 (39.4)	
**Mother's level of education**	No education	35 (47.9)	38 (52.1)	*p**	38 (52.1)	35 (47.9)	*NS*	36 (49.3)	37 (50.7)	*NS*	44 (60.3)	29 (39.7)	*NS*	48 (65.8)	25 (34.2)	*p**	46 (63.0)	27 (37.0)	*p**
	Primary	175 (43.9)	224 (56.1)		247 (61.9)	152 (38.1)		231 (57.9)	168 (42.1)		267 (66.9)	132 (33.1)		235 (58.9)	164 (41.1)		238 (59.6)	161 (40.4)	
	Complementary	364 (50.4)	358 (49.6)		423 (58.6)	299 (41.4)		361 (50.0)	361 (50.0)		491 (68.0)	231 (32.0)		473 (65.5)	249 (34.5)		421 (58.3)	301 (41.7)	
	Secondary	415 (58.9)	290 (41.1)		434 (61.6)	271 (38.4)		376 (53.3)	329 (46.7)		489 (69.4)	216 (30.6)		469 (66.5)	236 (33.5)		450 (63.8)	255 (36.2)	
	Undergraduate	238 (54.6)	198 (45.4)		267 (61.2)	169 (38.8)		222 (50.9)	214 (49.1)		302 (69.3)	134 (30.7)		321 (73.6)	115 (26.4)		233 (53.4)	203 (46.6)	
	Graduate	491 (54.1)	416 (45.9)		564 (62.2)	343 (37.8)		496 (54.7)	411 (45.3)		640 (70.6)	267 (29.4)		653 (72.0)	254 (28.0)		537 (59.2)	370 (40.8)	
**Mother's marital status**	Single	23 (23.7)	74 (76.3)	*p**	72 (74.2)	25 (25.8)	*NS*	80 (82.5)	17 (17.5)	*p**	79 (81.4)	18 (18.6)	*p**	69 (71.1)	28 (28.9)	*NS*	67 (69.1)	30 (30.9)	*NS*
	Married	1641 (53.9)	1404 (46.1)		1841 (60.5)	1204 (39.5)		1586 (52.1)	1459 (47.9)		2086 (68.5)	959 (31.5)		2069 (67.9)	976 (32.1)		1802 (59.2)	1243 (40.8)	
	Divorced	36 (50.7)	35 (49.3)		43 (60.6)	28 (39.4)		35 (49.3)	36 (50.7)		46 (64.8)	25 (35.2)		42 (59.2)	29 (40.8)		40 (56.3)	31 (43.7)	
	Widow	18 (62.1)	11 (37.9)		17 (58.6)	12 (41.4)		21 (72.4)	8 (27.6)		22 (75.9)	7 (24.1)		19 (65.5)	10 (34.5)		16 (55.2)	13 (44.8)	
**Father's occupation**	No Work	2 (25.0)	6 (75.0)	*p**	6 (75.0)	2 (25.0)	*NS*	7 (87.5)	1 (12.5)	*NS*	7 (87.5)	1 (12.5)	*p**	5 (62.5)	3 (37.5)	*p**	4 (50.0)	4 (50.0)	*NS*
	Housewife	2 (40.0)	3 (6.0)		4 (80)	1 (20.0)		3 (60.0)	2 (40.0)		3 (60.0)	2 (40.0)		4 (80.0)	1 (20.0)		3 (60.0)	2 (40.0)	
	Student	4 (36.4)	7 (63.6)		10 (90.9)	1 (9.1)		4 (36.4)	7 (63.6)		8 (72.7)	3 (27.3)		8 (72.7)	3 (27.3)		5 (45.5)	6 (54.5)	
	Employed	760 (56.5)	585 (43.5)		823 (61.2)	522 (38.8)		740 (55.0)	605 (45.0)		940 (69.9)	405 (30.1)		954 (70.9)	391 (29.1)		782 (58.1)	563 (41.9)	
	Self-employed	595 (53.6)	515 (46.4)		660 (59.5)	450 (40.5)		560 (50.5)	550 (49.5)		742 (66.8)	368 (33.2)		724 (65.2)	386 (34.8)		664 (59.8)	446 (40.2)	
	Unemployed	111 (52.1)	102 (47.9)		122 (57.3)	91 (42.7)		108 (50.7)	105 (49.3)		138 (64.8)	75 (35.2)		122 (57.3)	91 (42.7)		118 (55.4)	95 (44.6)	
	Retired	64 (54.2)	54 (45.8)		65 (55.1)	53 (44.9)		65 (55.1)	53 (44.9)		76 (64.4)	42 (35.6)		84 (71.2)	34 (28.8)		73 (61.9)	45 (38.1)	
	Disabled	8 (47.1)	9 (52.9)		8 (47.1)	9 (52.9)		9 (52.9)	8 (47.1)		11 (64.7)	6 (35.3)		11 (64.7)	6 (35.3)		10 (58.8)	7 (41.2)	
	Health field	16 (45.7)	19 (54.3)		19 (54.3)	16 (45.7)		21 (60.0)	14 (40.0)		16 (45.7)	19 (54.3)		28 (80.0)	7 (20.0)		19 (54.3)	16 (45.7)	
	Other	93 (39.7)	141 (60.3)		154 (65.8)	80 (34.2)		116 (49.6)	118 (50.4)		175 (74.8)	59 (25.2)		154 (65.8)	80 (34.2)		143 (61.1)	91 (38.9)	
**Father's level of education**	No education	69 (52.3)	63 (47.7)	*NS*	75 (56.8)	57 (43.2)	*NS*	75 (56.8)	57 (43.2)	*NS*	79 (59.8)	53 (40.2)	*NS*	88 (66.7)	44 (33.3)	*p**	80 (60.6)	52 (39.4)	*NS*
	Primary	291 (49.2)	301 (50.8)		345 (58.3)	247 (41.7)		320 (54.1)	272 (45.9)		392 (66.2)	200 (33.8)		380 (64.2)	212 (35.8)		344 (58.1)	248 (41.9)	
	Complementary	500 (52.4)	455 (47.6)		578 (60.5)	377 (39.5)		490 (51.3)	465 (48.7)		656 (68.7)	299 (31.3)		598 (62.6)	357 (37.4)		566 (59.3)	389 (40.7)	
	Secondary	401 (57.0)	302 (43.0)		441 (62.7)	262 (37.3)		378 (53.8)	325 (46.2)		489 (69.6)	214 (30.4)		498 (70.8)	205 (29.2)		426 (60.6)	277 (39.4)	
	Undergrad	144 (50.9)	139 (49.1)		176 (62.2)	107 (37.8)		153 (54.1)	130 (45.9)		204 (72.1)	79 (27.9)		215 (76.0)	68 (24.0)		163 (57.6)	120 (42.4)	
	Graduate	252 (55.6)	201 (44.4)		275 (60.7)	178 (39.3)		238 (52.5)	215 (47.5)		316 (69.8)	137 (30.2)		331 (73.1)	122 (26.9)		256 (56.5)	197 (43.5)	
**Father's marital status**	Single	12 (26.1)	34 (73.9)	*p**	34 (73.9)	12 (26.1)	*NS*	39 (84.8)	7 (15.2)	*p**	39 (84.8)	7 (15.2)	*NS*	34 (73.9)	12 (26.1)	*NS*	25 (54.3)	21 (45.7)	*NS*
	Married	1624 (53.6)	1407 (46.4)		1829 (60.3)	1202 (39.7)		1592 (52.5)	1439 (47.5)		2067 (68.2)	964 (31.8)		2048 (67.6)	983 (32.4)		1789 (59.0)	1242 (41.0)	
	Divorced	18 (50.0)	18 (50.0)		23 (63.9)	13 (36.1)		21 (58.3)	15 (41.7)		26 (72.2)	10 (27.8)		25 (69.4)	11 (30.6)		19 (52.8)	17 (47.2)	
	Widow	3 (60.0)	2 (40.0)		4 (80.0)	1 (20.0)		2 (40.0)	3 (60.0)		4 (80.0)	1 (20.0)		3 (60.0)	2 (40.0)		2 (40.0)	3 (60.0)	
**Medical History**	No	539 (47.3)	600 (52.7)	*p**	705 (61.9)	434 (38.1)	*NS*	611 (53.6)	528 (46.4)	*NS*	809 (71.0)	330 (29.0)	*p**	795 (69.8)	344 (30.2)	*NS*	644 (56.5)	495 (43.5)	*p**
	Yes	637 (57.9)	464 (42.1)		657 (59.7)	444 (40.3)		601 (54.6)	500 (45.4)		729 (66.2)	372 (33.8)		737 (66.9)	364 (33.1)		678 (61.6)	423 (38.4)	
**Ongoing treatment**	No	868 (51.7)	812 (48.3)	*NS*	1033 (61.5)	647 (38.5)	*NS*	920 (54.8)	760 (45.2)	*NS*	1139 (67.8)	541 (32.2)	*p**	1129 (67.2)	551 (32.8)	*NS*	984 (58.6)	696 (41.4)	*NS*
	Yes	161 (52.8)	144 (47.2)		200 (65.6)	105 (34.4)		174 (57.0)	131 (43.0)		236 (77.4)	69 (22.6)		211 (69.2)	94 (30.8)		176 (57.7)	129 (42.3)	
**Child visits: pediatrician**	No	198 (48.1)	214 (51.9)	*NS*	238 (57.8)	174 (42.2)	*NS*	212 (51.5)	200 (48.5)	*NS*	260 (63.1)	152 (36.9)	*p**	264 (64.1)	148 (35.9)	*NS*	252 (61.2)	160 (38.8)	*NS*
	Yes	825 (53.0)	732 (47.0)		986 (63.3)	571 (36.7)		869 (55.8)	688 (44.2)		1105 (71.0)	452 (29.0)		1065 (68.4)	492 (31.6)		898 (57.7)	659 (42.3)	
**Child visits: psychiatrist**	No	1683 (52.7)	1513 (47.3)	*NS*	1931 (60.4)	1265 (39.6)	*NS*	1676 (52.4)	1520 (47.6)	*p**	2207 (69.1)	989 (30.9)	*NS*	2149 (67.2)	1047 (32.8)	*NS*	1885 (59.0)	1311 (41.0)	*NS*
	Yes	77 (53.8)	66 (46.2)		92 (64.3)	51 (35.7)		87 (60.8)	56 (39.2)		88 (61.5)	55 (38.5)		101 (70.6)	42 (29.4)		91 (63.6)	52 (36.4)	

Table S2. Sociodemographic and other factors affecting entities of extreme mismatch.

		**Oral cavities**			**Enlarged tonsils**			**Bullying**		
		**Mismatch**	**Match**		**Mismatch**	Match		Mismatch	Match	
**School type**	Public	536 (46.4)	620 (53.6)	p*	552 (47.8)	604 (52.2)	p*	332 (28.7)	824 (71.3)	p*
	Private	825 (37.1)	1399 (62.9)		798 (35.9)	1426 (64.1)		779 (35.0)	1445 (65.0)	
**Governorate**	Beirut	83 (44.4)	104 (55.6)	p*	89 (47.6)	98 (52.4)	p*	73 (39.0)	114 (61.0)	p*
	Mount Lebanon	270 (35.6)	488 (64.4)		291 (38.4)	467 (61.6)		241 (31.8)	517 (68.2)	
	North - Akkar	339 (42.5)	458 (57.5)		343 (43.0)	454 (57.0)		204 (25.6)	593 (74.4)	
	Bekaa, Baalbek Hermel	315 (44.2)	397 (55.8)		278 (39.0)	434 (61.0)		311 (43.7)	401 (56.3)	
	South	354 (38.2)	572 (61.8)		349 (37.7)	577 (62.3)		282 (30.5)	644 (69.5)	
**Age**	3 - 6 years	474 (35.3)	870 (64.7)	p*	487 (36.2)	857 (63.8)	p*	321 (23.9)	1023 (76.1)	p*
	7 - 9 years	429 (42.4)	583 (57.6)		461 (45.6)	551 (54.4)		389 (38.4)	623 (61.6)	
	10 - 12 years	458 (44.7)	566 (55.3)		402 (39.3)	622 (60.7)		401 (39.2)	623 (60.8)	
**Gender**	Male	660 (40.2)	980 (59.8)	NS	691 (42.1)	949 (57.9)	p*	564 (34.4)	1076 (65.6)	NS
	Female	701 (40.3)	1039 (59.7)		659 (37.9)	1081 (62.1)		547 (31.4)	1193 (68.6)	
**Family Income**	No income	119 (43.0)	158 (57.0)	p*	99 (35.7)	178 (64.3)	p*	100 (36.1)	177 (63.9)	p*
	<100$	530 (43.1)	701 (56.9)		562 (45.7)	669 (54.3)		418 (34.0)	813 (66.0)	
	100-300$	137 (41.3)	195 (58.7)		118 (35.5)	214 (64.5)		99 (29.8)	233 (70.2)	
	300-600$	320 (35.5)	582 (64.5)		348 (38.6)	554 (61.4)		338 (37.5)	564 (62.5)	
	600-900$	77 (33.9)	150 (66.1)		76 (33.5)	151 (66.5)		64 (28.2)	163 (71.8)	
	>900$	8 (29.6)	19 (70.4)		12 (44.4)	15 (55.6)		11 (40.7)	16 (59.3)	
**BMI**	Underweight	581 (71.2)	235 (28.8)	NS	531 (65.1)	285 (34.9)	NS	547 (67.0)	269 (33.0)	NS
	Normal weight	964 (71.8)	378 (28.2)		876 (65.3)	466 (34.7)		899 (67.0)	443 (33.0)	
	Overweight	797 (70.6)	332 (29.4)		746 (66.1)	383 (33.9)		733 (64.9)	396 (35.1)	
**Mother's occupation**	No Work	0 (0.0)	3 (100.0)	p*	1 (33.3)	2 (66.7)	p*	1 (33.3)	2 (66.7)	NS
	Housewife	930 (41.3)	1321 (58.7)		939 (41.7)	1312 (58.3)		748 (33.2)	1503 (66.8)	
	Student	5 (31.3)	11 (68.8)		6 (37.5)	10 (62.5)		7 (43.8)	9 (56.3)	
	Employed	198 (33.6)	391 (66.4)		186 (31.6)	403 (68.4)		190 (32.3)	399 (67.7)	
	Self employed	50 (37.3)	84 (62.7)		51 (38.1)	83 (61.9)		49 (36.6)	85 (63.4)	
	Unemployed	34 (38.6)	54 (61.4)		35 (39.8)	53 (60.2)		29 (33.0)	59 (67.0)	
	Retired	5 (55.6)	4 (44.4)		7 (77.8)	2 (22.2)		5 (55.6)	4 (44.4)	
	Disabled	2 (100.0)	0 (0.0)		1 (50.0)	1 (50.0)		0 (0.0)	2 (100.0)	
	Health field	25 (32.9)	51 (67.1)		20 (26.3)	56 (73.7)		25 (32.9)	51 (67.1)	
	Other	16 (48.5)	17 (51.5)		7 (21.2)	26 (78.8)		7 (21.2)	26 (78.8)	
**Mother's level of education**	No education	38 (52.1)	35 (47.9)	p*	39 (53.4)	34 (46.6)	p*	20 (27.4)	53 (72.6)	NS
	Primary	188 (47.1)	211 (52.9)		208 (52.1)	191 (47.9)		120 (30.1)	279 (69.9)	
	Complementary	307 (42.5)	415 (57.5)		322 (44.6)	400 (55.4)		241 (33.4)	481 (66.6)	
	Secondary	262 (37.2)	443 (62.8)		278 (39.4)	427 (60.6)		239 (33.9)	466 (66.1)	
	Undergraduate	177 (40.6)	259 (59.4)		166 (38.1)	270 (61.9)		138 (31.7)	298 (68.3)	
	University graduate	324 (35.7)	583 (64.3)		274 (30.2)	633 (69.8)		307 (33.8)	600 (66.2)	
**Mother's marital status**	Single	55 (56.7)	42 (43.3)	p*	67 (69.1)	30 (30.9)	p*	30 (30.9)	67 (69.1)	NS
	Married	1205 (39.6)	1840 (60.4)		1185 (38.9)	1860 (61.1)		991 (32.5)	2054 (67.5)	
	Divorced	26 (36.6)	45 (63.4)		27 (38.0)	44 (62.0)		31 (43.7)	40 (56.3)	
	Widow	10 (34.5)	19 (65.5)		8 (27.6)	21 (72.4)		13 (44.8)	16 (55.2)	
**Father's occupation**	No Work	2 (25.0)	6 (75.0)	NS	3 (37.5)	5 (62.5)	NS	1 (12.5)	7 (87.5)	NS
	Housewife	4 (80.0)	1 (20.0)		2 (40.0)	3 (60.0)		1 (20.0)	4 (80.0)	
	Stufent	5 (45.5)	6 (54.5)		3 (27.3)	8 (72.7)		5 (45.5)	6 (54.5)	
	Employed	503 (37.4)	842 (62.6)		503 (37.4)	842 (62.6)		418 (31.1)	927 (68.9)	
	Self-employed	443 (39.9)	667 (60.1)		454 (40.9)	656 (59.1)		386 (34.8)	724 (65.2)	
	Unemployed	98 (46.0)	115 (54.0)		81 (38.0)	132 (62.0)		75 (35.2)	138 (64.8)	
	Retired	42 (35.6)	76 (64.4)		39 (33.1)	79 (66.9)		42 (35.6)	76 (64.4)	
	Disabled	9 (52.9)	8 (47.1)		6 (35.3)	11 (64.7)		7 (41.2)	10 (58.8)	
	Health field	12 (34.3)	23 (65.7)		12 (34.3)	23 (65.7)		12 (34.3)	23 (65.7)	
	Other	96 (41.0)	138 (59.0)		98 (41.9)	136 (58.1)		79 (33.8)	155 (66.2)	
	No education	67 (50.8)	65 (49.2)	p*	49 (37.1)	83 (62.9)	p*	43 (32.6)	89 (67.4)	NS

**Father's level of education**	Primary	261 (44.1)	331 (55.9)		284 (48.0)	308 (52.0)		194 (32.8)	398 (67.2)	
	Complementary	374 (39.2)	581 (60.8)		396 (41.5)	559 (58.5)		301 (31.5)	654 (68.5)	
	Secondary	276 (39.3)	427 (60.7)		255 (36.3)	448 (63.7)		244 (34.7)	459 (65.3)	
	Undergrad	107 (37.8)	176 (62.2)		89 (31.4)	194 (68.6)		88 (31.1)	195 (68.9)	
	Graduate	145 (32.0)	308 (68.0)		147 (32.5)	306 (67.5)		160 (35.3)	293 (64.7)	
**Father's marital status**	Single	28 (60.9)	18 (39.1)	p*	30 (65.2)	16 (34.8)	p*	13 (28.3)	33 (71.7)	NS
	Married	1191 (39.3)	1840 (60.7)		1174 (38.7)	1857 (61.3)		1004 (33.1)	2027 (66.9)	
	Divorced	10 (27.8)	26 (72.2)		12 (33.3)	24 (66.7)		12 (33.3)	24 (66.7)	
	Widow	1 (20.0)	4 (80.0)		4 (80.0)	1 (20.0)		1 (20.0)	4 (80.0)	
**Medical History**	No	462 (40.6)	677 (59.4)	NS	480 (42.1)	659 (57.9)	NS	350 (30.7)	789 (69.3)	NS
	Yes	453 (41.1)	648 (58.9)		434 (39.4)	667 (60.6)		381 (34.6)	720 (65.4)	
**Child with ongoing treatment**	No	706 (42.0)	974 (58.0)	p*	654 (38.9)	1026 (61.1)	NS	526 (31.3)	1154 (68.7)	NS
	Yes	101 (33.1)	204 (66.9)		115 (37.7)	190 (62.3)		99 (32.5)	206 (67.5)	
**Child visits to the pediatrician/doctor**	No	165 (40.0)	247 (60.0)	NS	153 (37.1)	259 (62.9)	NS	147 (35.7)	265 (64.3)	p*
	Yes	635 (40.8)	922 (59.2)		613 (39.4)	944 (60.6)		470 (30.2)	1087 (69.8)	
**Previous child visits to the psychiatrist**	No	1287 (40.3)	1909 (59.7)	NS	1262 (39.5)	1934 (60.5)	NS	1020 (31.9)	2176 (68.1)	p*
	Yes	49 (34.3)	94 (65.7)		59 (41.3)	84 (58.7)		71 (49.7)	72 (50.3)	

p* = $p < 0.05$
NS = Non-significant p value > 0.05
